# DMET-Analyzer: automatic analysis of Affymetrix DMET Data

**DOI:** 10.1186/1471-2105-13-258

**Published:** 2012-10-05

**Authors:** Pietro Hiram Guzzi, Giuseppe Agapito, Maria Teresa Di Martino, Mariamena Arbitrio, Pierfrancesco Tassone, Pierosandro Tagliaferri, Mario Cannataro

**Affiliations:** 1 Department of Medical and Surgical Sciences, Magna Graecia University of Catanzaro, Catanzaro, Italy; 2 Medical Oncology Unit and Tommaso Campanella Cancer Center, Magna Graecia University of Catanzaro, Catanzaro, Italy; 3 Institute of Neurological Science (ISN-CNR), UOS of Pharmacology, Roccelletta di Borgia, Catanzaro, Italy

## Abstract

**Background:**

Clinical Bioinformatics is currently growing and is based on the integration of clinical and omics data aiming at the development of personalized medicine. Thus the introduction of novel technologies able to investigate the relationship among clinical states and biological machineries may help the development of this field. For instance the Affymetrix DMET platform (drug metabolism enzymes and transporters) is able to study the relationship among the variation of the genome of patients and drug metabolism, detecting SNPs (Single Nucleotide Polymorphism) on genes related to drug metabolism. This may allow for instance to find genetic variants in patients which present different drug responses, in pharmacogenomics and clinical studies. Despite this, there is currently a lack in the development of open-source algorithms and tools for the analysis of DMET data. Existing software tools for DMET data generally allow only the preprocessing of binary data (e.g. the DMET-Console provided by Affymetrix) and simple data analysis operations, but do not allow to test the association of the presence of SNPs with the response to drugs.

**Results:**

We developed DMET-Analyzer a tool for the automatic association analysis among the variation of the patient genomes and the clinical conditions of patients, i.e. the different response to drugs. The proposed system allows: (i) to automatize the workflow of analysis of DMET-SNP data avoiding the use of multiple tools; (ii) the automatic annotation of DMET-SNP data and the search in existing databases of SNPs (e.g. dbSNP), (iii) the association of SNP with pathway through the search in PharmaGKB, a major knowledge base for pharmacogenomic studies. DMET-Analyzer has a simple graphical user interface that allows users (doctors/biologists) to upload and analyse DMET files produced by Affymetrix DMET-Console in an interactive way. The effectiveness and easy use of DMET Analyzer is demonstrated through different case studies regarding the analysis of clinical datasets produced in the University Hospital of Catanzaro, Italy.

**Conclusion:**

DMET Analyzer is a novel tool able to automatically analyse data produced by the DMET-platform in case-control association studies. Using such tool user may avoid wasting time in the manual execution of multiple statistical tests avoiding possible errors and reducing the amount of time needed for a whole experiment. Moreover annotations and the direct link to external databases may increase the biological knowledge extracted. The system is freely available for academic purposes at: https://sourceforge.net/projects/dmetanalyzer/files/

## Background

Nowadays the tight collaboration among molecular biologists, medical doctors and computer scientists resulted in the development of novel research areas in which they share their experiences and know-how. Thus the classical bioinformatics field devoted to the investigation of biological data has moved towards the clinical scenario [1,2].

A main research direction of this area is represented by the possibility to integrate biological and clinical data, e.g. through the integration of omics data into Electronic Patient Records. The combined use of genomics, proteomics, and clinical data may thus improve healthcare management allowing the development of novel therapies that are customised to the patients on the basis of their own characteristics. The rationale is that the exhaustive comprehension of biological systems may enable the development of the so-called personalized medicine, because the response to a treatment is determined by the characteristics of the genome of each individual [3]. This may enable the development of therapies and drugs that are targeted to a specific patient improving the effectiveness of drugs themselves [4,5].

The recent introduction of new technologies, such as the Affymetrix SNP 5.0, SNP 6.0, and DMET ( http://www.affymetrix.com) and the Illumina Genotyping ( http://www.Illumina.com), have enabled the high-throughput analysis of the genomes of patients. As a consequence large-scale studies of genetic variations in human are made possible at a relatively moderate costs yielding a big impact in the Translational Medicine. In a clinical scenario such a possibility has enabled the design of studies aiming at the identification of the genomic variants that may correlate with different classes of phenotypes, such as diseases, or response to drugs, e.g.in pharmacogenomics experiments [6-9].

Each individual has a unique sequence of DNA that determines his/her characteristics. Differences can be measured in terms of substitutions of bases in the same position. Research focused particularly in the substitution of a single base that occurs in a small subset of the population. These mutations, also referred to as single nucleotide polymorphisms (SNP’s), are usually defined as a stable substitution of a single base with a frequency of more than 1% in at least one population. Let us consider the short sequences ATGT and ACGT, a base change occurs at position 2 and is denoted by “T/C” [10,11].

Many works have demonstrated a correlation between the presence of SNPs and the development of diseases, and more recently the effectiveness of drugs [12,13]. Thus the presence (or the absence) of specific SNPs may be used as a clinical marker for the prediction of drug effectiveness, foreseeing the response of individuals with different SNPs to drugs.

Such sub-field of genomics, also known as pharmacogenomics, concerns the study of variations in genes responsible for the metabolism of drugs. Moreover, pharmacogenomics also focuses on the investigation of adverse drug reactions (ADR) that occur most frequently when a drug has a narrow therapeutic index. The therapeutic index is a measure of the amount of drug that may cause lethal effect. When a drug has a narrow therapeutic index, this means that there exists little difference between the lethal and the therapeutic dose.

Pharmacogenomics experiments (as deeply discussed in next Section), involve the selection of a candidate cohort of population, the gene sequencing and the individuation of SNPs by using microarray technology and computational analysis. The DMET (drug metabolism enzymes and transporters) Plus Premier Pack is a novel microarray assay developed by Affymetrix^a^ designed specifically to test drug metabolism associations [14] in pharmacogenomics case-control study.

DMET is able to genotypize function variants in a defined set that comprises 225 ADME-related genes, i.e. genes known to be related to drug absorption, distribution, metabolism and excretion (ADME) [15-17]. Different recent works demonstrated the roles of genetic variations in ADME genes in association with the heterogeneity in drug treatment effects. For instance, Li et al. [15] demonstrated in a systematic way that polymorphisms in ADME genes are correlated to the difference in drug responses. In that work authors systematically tested ADME genes, then analyzed the polimorphisms demonstrating the association with response to drugs.

In two works of Di Martino et al. [18,19], the DMET platform has been used to evidentiate polimorphisms related to toxicity of drugs in two different cancer types. The workflow of these works is the same: data produced by DMET platform are manually mined by scientists. The use of DMET-Analyzer, as explained in following section, may automatize the analysis of these data, resulting in obtaining the same results in less time.

Data produced by DMET platform must be preprocessed and analyzed in order to find correlation between the presence/absence of SNPs and the status of samples (e.g. type of drug treatment). To the best of our knowledge, existing software tools, e.g. the DMET Console platform, generally allow only the preprocessing of binary data and simple data analysis operations, but do not allow to test the association of the presence of SNPs with the response to drugs. Consequently, researches have to export and manually process SNPs tables produced by the DMET Console. The discovery of statistically significant associations requires the use of external tools (e.g. statistical softwares) and the manual execution of multiple tests.

An association study represents an exciting application scenario for clinical bioinformatics [20]. In fact there is the need for introducing both methodologies and software tools supporting all the phases of the experiments. During the design phase, accurate studies have to determine both the right population size and the sampling strategies avoiding: (i) that final results may be biased of either the sampling strategy or the sample size, (ii) that money is wasted by the effectuation of meaningless experiments.

In this paper we present DMET-Analyzer, a tool for the automatic statistics test of the association between SNPs and examined sample conditions. DMET Analyzer is a platform-independent software built in *Java* that supports the statistical analysis of DMET data. It has a simple graphical user interface that allows users to upload and analyse DMET files produced by DMET Console in an interactive way. Considering the usual workflow of a pharmacogenomics experiment, the proposed tool receives as input DMET data produced by the Affymetrix platform and produces as output a list of candidate SNPs together with their biological and pharmacogenomics interpretations as stored in main public databases.

### DMET Case Control Data Analysis workflow

**Figure 1:**
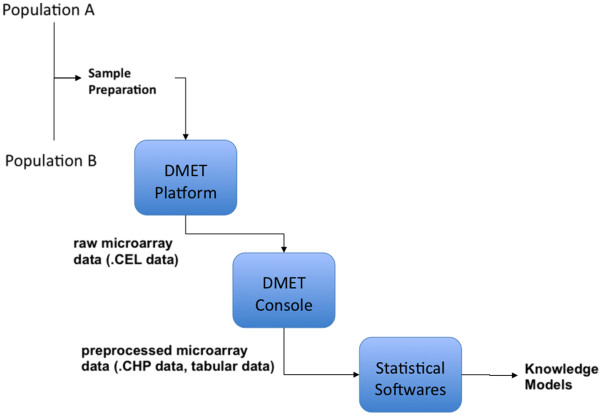
**Workflow of a clinical bioinformatics experiment from the sample collection to the data analysis.** Workflow of data in a typical experiment

A typical workflow of a case-control association study performed by using the DMET platform. involves the following steps, as depicted in Figure 1: 

**Sample collection and DMET chip preparation:** in this phase biological samples are collected and treated to perform microarray experiments; the Affymetrix DMET chip allows the investigation of 1936 different nucleotides that present possible variants as stored in SNP databases, each one representing a portion of the genome having a role in drug metabolism;

**DMET microarray experiments**, this phase produces first raw microarray data (.CEL data);

**DMET data preprocessing**: raw data (.CEL) produced by the instrument are further preprocessed by the DMET console software that produces as output single preprocessed files (.CHP) for each sample or the whole dataset representing all the samples (usually tabular data); the DMET Console software produces a table containing, for each nucleotide and for each sample, the detected SNP or a NoCall value (where NoCall means that the platform has not revealed the nucleotide with a sufficient confidence).

**SNPs detection:**, this phase statistically analyses SNP data producing as output knowledge models (e.g. a binary classifier built on top of the SNP distribution among classes) or statistical models that help to find significant SNPs. The selection of the statistical test span over a broad range, from simple Fisher’s tests that analyse the significance of a single SNP to complex tests that analyse those of a set of SNPs.

## Implementation

DMET-Analyzer tool supports the visualization of the SNPs detected on the entire dataset as a heatmap to give an immediate visual feedback to the user. It implements a Hardy-Weinberg equilibrium calculator that can be used for testing the genetic model. It is able to automatically read the class assigned to each sample (patient) that can be provided in the header row of the DMET dataset. Finally, it annotates significant SNPs with information provided by Affymetrix libraries and with links to the dbSNP database (for basic information about SNPs) and to the PharmaGKB pharmacogenomics knowledge base, giving various information (e.g. pathways) related to pharmacogemomics.

### DMET Analyzer: a tool for DMET Data Analysis

In this section we describe main functions of DMET Analyzer. The tool sits in the middle of a typical workflow of an association-study experiment aiming at the identification of the discriminative SNPs among two classes as depicted, in Figure 1, and consequently main functions of our software are: 

**Loading and Visualization of Data produced by DMET console:** DMET Analyzer currently is able to parse information encoded as excel data files as well as tab delimited files. User in this way may also prepare his/her own dataset, e.g. merging together samples coming from different experimental batches. The structure of the input file is described in Table 1. The software is able to find the class-labels directly from the input files. For instance, the classes may be healthy-diseased or the kind of response to drugs, e.g. toxicity or not-toxicity. It is possible to visualize the SNP distribution of the dataset in order to enable a fast analysis of data themselves.

**Analysis of Variants:** DMET Analyzer automatically selects the relevant SNPs. The current version of DMET Analyzer verifies, for each SNP, the association among the presence of SNP and the classes yet determined through the use of the well known Fisher’s test. Fisher’s test has been chosen because literature contains examples of pharmacogenomics studies containing few samples (less than 20) so this test represents a good choice [21].Moreover, two multiple test corrections are available (Bonferroni and False Discovery Rate) in order to improve the statistical significance of results. For each SNP it is possible to analyse the linkage disequilibrium by using a Hardy-Weinberg calculator embedded into DMET-Analyzer.The current version of DMET-Analyzer contains a simple implementation of the Pearson’s chi-square test to calculate the deviation from the Hardy-Weinberg equilibrium for bi-allelic probesets. The calculator is manual, so the user has to insert the observed allele frequencies for the Homozygote reference, the Homozygote variant and the Heterozygote, and the significance level of the test. The calculator will estimate the deviation from the equilibrium and will test the hypothesis that such deviation is significant.

**Annotation of Data:** Finally, for each SNP it is possible to access both annotations provided by Affymetrix and the dbSNP [22] to explain the biological finding of results.

**Pharmacogenomics Interpretation:** For each analyzed SNP, it is possible to obtain additional information stored in the Pharmacogenomics Knowledge Base [23]. It is also possible to obtain additional information about the analyzed SNPs and their clinical interpretation associated with drug response, as well as drug dosing guidelines, drug-centered pathways, and relationships among genes, drugs and diseases.

**Table 1:** DMET data format

**Probes**	***Subjec***_***t*1**_	***Subjec***_***t*2**_	***Subjec***_***t*3**_	***Subjec***_***t*4**_
*Prob*_*e*1_	C/C	C/C	T/T	T/T
*Prob*_*e*2_	G/C	C/C	-/T	A/A
*Prob*_*e*3_	C/T	C/T	C/T	C/T
*Prob*_*en*_	G/G	A/G	G/G	G/G

In this table is represented a possible Example of input table, produced at the end of DMET-Console workflow. Where each row represents a probe identified by its own identifier, and each column represents a subject represented by its own identifier. File must contain in the first row a list of specific identifiers: the probe_set identifiers in the first column and the identifiers of subjects, in the subsequent ones. The *cell ( i,j)* contains the allele belonging at *i-th *subject into the *j-th* probe_set, identified in the previous analysis.

#### DMET Analyzer Implementation

DMET Analyzer is a platform-independent application and it is entirely implemented using the Java programming language^b^.

#### DMET Analyzer User Interface

DMET Analyzer provides a simple Graphical User Interface allowing the user an easy access to the tool functionalities. After launching the software, a simple user interface enables the user to load data files into the system. After that the loading is completed, data are arranged automatically in tabular form and samples are automatically assigned to classes specified on the input files.

Now it is possible to begin the analysis step starting the execution of all statistical tests. In order to avoid wasting time on the calculation of meaningless tests, DMET Analyzer employs an optional optimization step that enable the calculation of Fisher’s tests only for probes whose SNPs distributions presents a difference greater than a threshold. It should be noted that we also offer the possibility to calculate all the tests without this optimization step, even if the optimization step has not introduced bias nor eliminated useful SNPs.

Moreover, user can specify a subset of rows to test.

## Results and discussion

### Algorithm of analysis in DMET Analyzer

This section presents the analysis algorithm implemented into DMET Analyzer. The algorithm takes as input a data matrix representing the detected SNPs for each patient and the classes to which a patient belongs to. Table 1 represents an example of such data. The analysis algorithm iteratively considers each row and analyses the frequency distribution of each SNP. For each symbol a Fisher’s test is performed and statistically significant differences among classes are reported. Let us consider, for instance, Table 1 that represents a compact example of data produced by DMET console. First row represents the names of the samples while first column contains the identifier of SNPs. A generic element (*i*,*j*) contains the *i*−*th* identified SNP in the *j*−*th* sample, so it has the form *X*/*Y*, where *X*,*Y*∈{*A*,*T*,*C*,*G*,−}. Now let us suppose, without loss of generality, that the first two columns belong to class *A* while the remaining ones belong to class *B*. It is evident that distribution of *Prob*_*e*1_is clearly different among two classes. The analysis algorithm iteratively and automatically test such differences for each SNP. The output of such an algorithm is a list of SNPs candidates and the related p-values whose distribution is different among classes. Such information need to be further tested or validated through integration with other biological information or with other wet lab experiments. For each SNP the annotations provided by Affymetrix are also available and, for further investigations, the system allows the automatic search into the dbSNP database or the PharmaGKB.

### Analysis of data in DMET-Analyzer

As proof-of-principle we tested the ability of DMET Analyzer to find statistically relevant SNPs on real data and for evaluating the performances we used some synthetic datasets. We built some synthetic datasets containing the same number of probes as real DMET data and an increasing number of samples grouped in two classes. We populated these data with randomly distributions of SNPs and significantly different distributions of SNPs. Tests revealed the ability of DMET Analyzer to recognize statistically different SNPs, from the other ones. These datasets are available for test on the DMET Analyzer web site.

**Figure 2:**
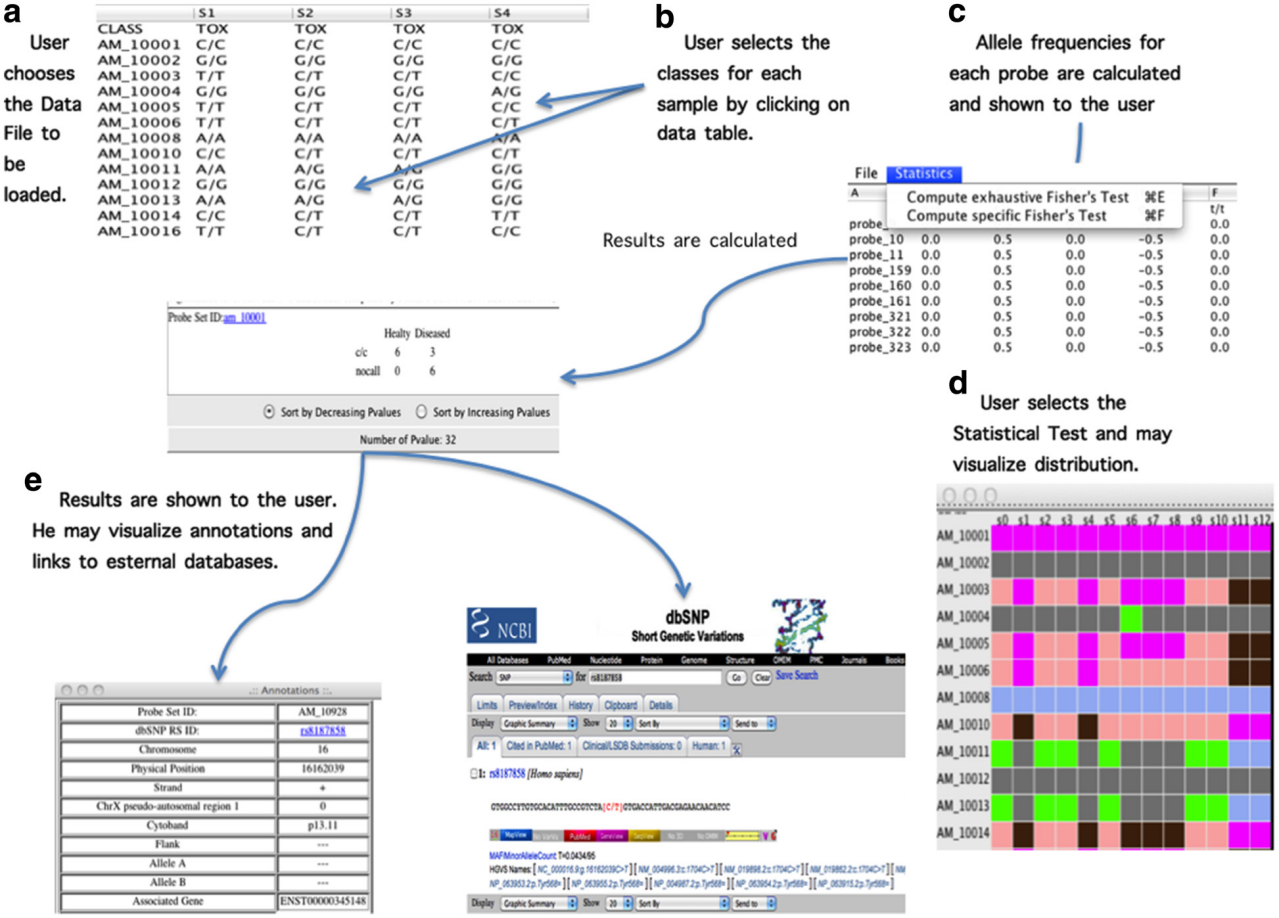
**Workflow of an experiment of analysis through the software.** Figure shows the workflow of execution of a typical analysis. Initially user loads data into the software as depicted in the upper left corner of Figure 2(**a**). Then user has to attribute the right class to each sample (Figure 2**b**) and to choose the analysis method Figure 2(**c**). The software calculates the allele frequencies for each allele and for each probe. At this point DMET Analyzer calculates the Fisher’s-tests and finally it shows the results in a new window in which probes may be sorted alphabetically or by p-value as depicted in Figure 2(**d**). User can select a SNP in this table and may visualize annotation data by just clicking on the SNP identifier as depicted in Figure 2(**e**). Analogously, user may visualize the distribution of variants using the embedded visualizer as evidenced in Figure 2(**f**)

In order to process data using DMET Analyzer user has to load data into the software. Data can be stored as a textual file or in a Excel data file (in this example we use an Excel data file). After that the loading has been completed DMET Analyzer shows the data table to the user as depicted in Figure 2(a) (first row contains the sample identifiers, first column contains the probe identifiers where each cell contain the identified alleles). User has to attribute the right class to each sample (Figure 2-b). At this point user can start the preprocessing by selecting the proper function in the upper menu. Actually DMET Analyzer offers the calculation of the exact Fisher’s-test for all the alleles or for a specified set of alleles and three possible method for calculating the p-value (no correction, Bonferroni Corrected and FDR). In both cases user has to select the two classes of the dataset and the p-value calculation method as depicted in Figure 2(c). At this point DMET Analyzer calculates the Fisher’s-tests, and finally it shows the results in a new window in which probes may be sorted alphabetically or by p-value as depicted in Figure 2(d). User can select a SNP in this table and may visualize annotation data by just clicking on the SNP identifier as depicted in Figure 2(e). Software offers both the annotations provided by Affymetrix and a link to external databases (dbSNP in the current version). DMET Analyzer also provides a compact visualization of data as depicted in Figure 2(g).

### Scalability and benchmark

**Figure 3:**
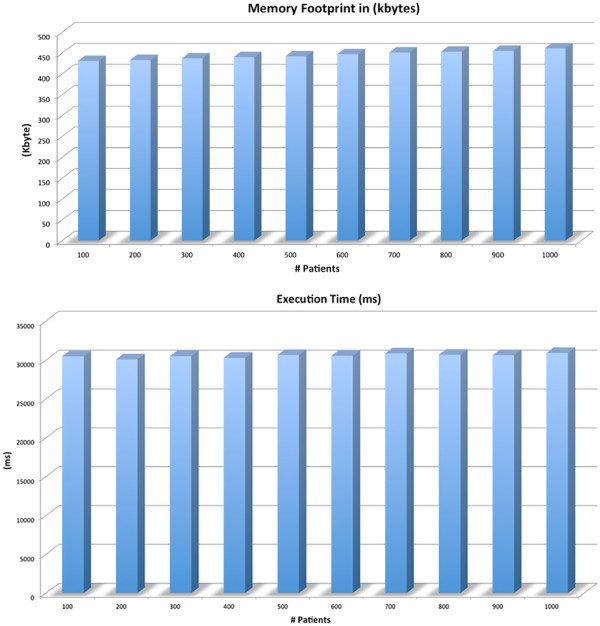
**Memory Occupancy and Execution Times.** Figure shows the execution time and the total amount of requested memory for a growing dimension of dataset. We performed these measures for different datasets considering ten datasets from 100 to 1000 patients increased by 100. Results show that the implementation of DMET Analyzer and the algorithmic choice enable the processing of this dataset requesting approximately the same time and the same memory for the execution (except for the initial loading of files)

Here we show the effectiveness of our approach showing the computation times, the memory occupancy and the scalability for a growing number of patients. It should be noted that our approach is based on the calculation of Fisher test for each SNP, then the performance are related to the number of computed tests and are independent from the number of patients. Figure 3 shows how the execution times for a growing number of patients from 100 to 1000 (typical numbers in genomics studies) are substantially unvaried while the memory occupancy remains almost unaffected by the high number of patients. Experiments are conducted on a Mac OS X 10.7.2, equipped with an Intel Core 2 Duo 2.2 Ghz processor, 4Gb of RAM memory and Java (TM) SE Runtime Environment.

### Analysis of real DMET Data

The effectiveness of the DMET Analyzer approach has been tested by the Bioinformatics Laboratory and the Tommaso Campanella Cancer Center [24,25]. In particular here we demonstrate as proof of concept the ability of DMET Analyzer to find the same results as those published in two works of Di Martino et al. [18,19], hereafter **Case Study 1**, and **Case Study 2**. In both works we took part in the data analysis performing the statistical tests in a manual way without the use of DMET Analyzer. Finally, we performed the analysis using our software, producing in a very smaller time the same results as those published in [18,19].

In **Case Study 1**[18], a cohort of 19 patients affected by multiple myeloma (MM) treated with aminobisphosphonate zoledronic acid (ZA) were enrolled in a case-control study. In particular nine patients presented osteonecrosis (ONJ) after the treatment and ten patients were the control ones.The study protocol was approved by our University Hospital Bioethical Committee and informed consent was obtained from each patient. The aim of the study was to investigate the association among specific SNPs and the adverse event ONJ induced by ZA. Results demonstrated the presence of eight SNPs that were related to ONJ. We obtained data from the local University Hospital after the deletion of personal identifier in compliance with the Italian law. Then we identified eight SNPs that were statistically associated with ONJ occurrence. We individuated a statistical difference among the distribution of SNPs: rs1152003, rs10893, rs4725373, rs1049793, rs2463437, rs903247, rs2468110, and rs2097937 (the same SNPs with same p-values were previously identified in the reference work).

In **Case Study 2**[19] a cohort of twenty six patients was used to investigate the genomic basis of the irinotecan-induced gastrointestinal (GI) toxicity by the novel drug-metabolizing enzyme and transporter (DMET) microarray genotyping platform. Patients who had undergone irinotecan-based chemotherapy enrolled experiencing a grade 3 gastrointestinal (GI) toxicity, control - matched patients without GI toxicity - study. We obtained data from the local university hospital after the deletion of personal identifier in compliance with the Italian law. We used DMET Analyzer to mine this data and we identified 3 SNPs mapping in ABCG1, ABCC5 and OATP1B1/SLCO1B1 transporter genes associated with GI toxicity: The homozygous genotype C/C in the ABCC5 gene (P=0.0022). The homozygous genotype G/G in the ABCG1 (P=0.0135) and the heterozygous genotype G/A in the OATP1B1/SLCO1B1 gene (P=0.0215). Results obtained using DMET Analyzer were the same as those in published work.

In conclusion, we were able to obtain the same results as published but performing in an automatic way the workflow of Figure 1 in a fraction of the time, and avoiding possible errors due to the manual investigation and processing of data.

### Related Work

The DMET platform has been recently introduced by Affymetrix, so there is a lack of methodologies of analysis and related tools. For the purposes of this work we discuss briefly the Affymetrix proprietary tools and we report some preliminary work of analysis. To the best of our knowledge, there is not a single tool able to implement all the steps of the workflow of analysis, especially for case-control association studies. Recent works discussing the use of such data, in fact, do not indicate a specific software for the statistical analysis but reuse existing platforms and specific procedures for translating the DMET datasets into a readable format [14,26]. In the rest of the section we briefly present a comparison of our software with respect to the state of the art tools. The comparison is made on the basis of a typical workflow of analysis and aims to evidence the functionalities of our tool and possible future improvements. Since our tool focuses on DMET data analysis, in the following we present a comparison with main tools for such analysis. Moreover in the Additional file 1 the interested reader may find a broader and deeper comparison with general tools for SNP analysis.

#### Comparison with existing Tools

##### Comparison with apt-dmet-genotype

**Figure 4:**
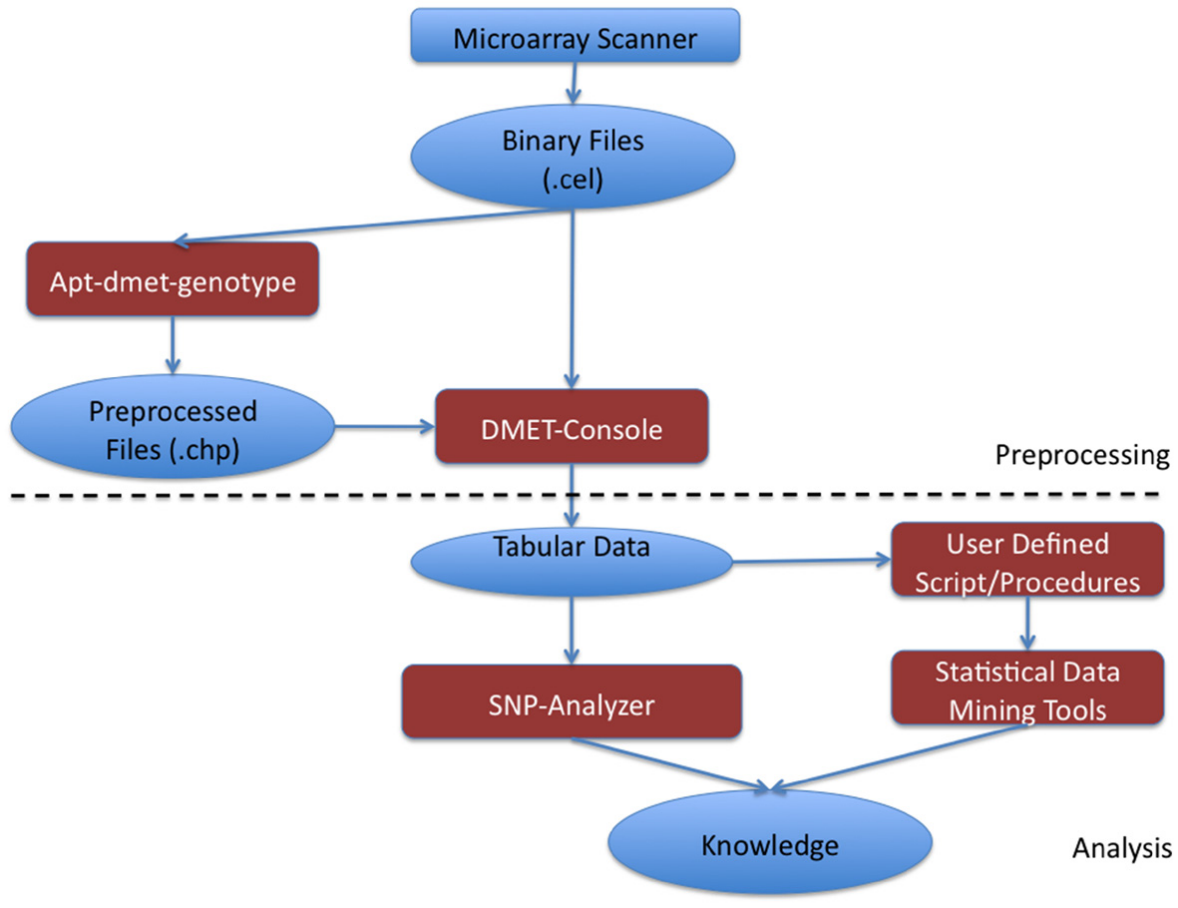
**Comparison with existing Tools.** Comparison of DMET Analyzer with respect to existing software tools considering a typical workflow of analysis. Data produced by the DMET platform may be preprocessed using apt-dmet-genotype. Then this data may be given as input to DMET-Console to be transformed into a format readable by other softwares. Diversely DMET console may perform these two steps. Then this data may be processed by statistical tools after some manual steps. Conversely our software is able to perform automatically all final steps

apt-dmet- genotype^a^ is a command-line software provided by Affymetrix that supports probe-set summarization of binary .CEL files, the management of resulting preprocessed files (.CHP). Considering the whole workflow of analysis described in this paper, the apt-dmet-genotype perform the first step, as depicted in Figure 4. It does not build the final tabular dataset containing the genotype call for all the probesets and all the samples. User consequently has to use the DMET Console to perform such phase. Compared to DMET Analyzer, apt-dmet-genotype presents some main differences: 

it lacks in the possibility of doing statistical analysis;

it is not extensible for the preprocessing of multivendor or user defined datasets;

it does not produce data that may be directly analysed.

On the other hand, the current version of DMET Analyzer lacks in the management of Affymetrix binary files. The adding of such functionalities may constitute the plan for future development of DMET Analyzer.

##### Comparison with DMET Console

DMET-Console^a^ is a GUI-based software provided by Affymetrix that supports probe-set summarization of binary .CEL files, the management of resulting preprocessed files (.CHP) and finally the building of a tabular dataset containing the genotype call for all the probesets and all the samples. It includes both genotypization and quality control algorithms as well as the possibility to make other analysis such as Copy Number Variations controls. Compared to DMET Analyzer, the Affymetrix Expression Console presents some main differences: 

it lacks in the possibility of doing statistical analysis

it is not extensible for the preprocessing of multivendor or user defined datasets;

On the other hand, the current version of DMET Analyzer lacks in quality control capabilities compared to DMET Console and in the possibility to manage directly Affymetrix binary files. Thus DMET-Console can be seen as a main datasource for our software.

## Conclusions

There exists an increasing interest in the scientific and medical community for the study of the impact of drugs on single patients and for the development of specific drugs for each patient. The rationale of this interest is based on the consideration that the response to the drugs is strictly related to the genomic differences, so the elucidation of these differences and their impact to the drug-response could unravel meaningful knowledge. Such discipline, known as pharmacogenomics, is a relatively novel field that is based on a technological platform for the investigation of the effect of drugs on single patients looking at their genomes. Although this is important, there is a lack of comprehensive tools able to perform all the steps of the workflow of analysis. In particular, while the preprocessing can be performed using freely available tools provided by the chip vendor, the subsequent analysis steps require the adaptation of data to existing tools.

In this paper we presented **DMET Analyzer**, a software platform for the analysis of such data able to read and extract significant SNP from Affymetrix DMET data. DMET Analyzer tool supports the visualization of the SNPs detected on the entire dataset as a heatmap to give an immediate visual feedback to the user. It implements a Hardy-Weinberg equilibrium calculator that can be used for testing the genetic model. It is able to automatically read the class assigned to each sample (patient) that can be provided in the header row of the DMET dataset. Finally, it annotates significant SNPs with information provided by Affymetrix libraries and with links to the dbSNP database (for basic information about SNPs) and to the PharmaGKB (Pharmacogenomics Knowledge Base), giving various information (e.g. pathways) related to pharmacogemomics. Additional file 2 presents a brief user guide.

## Availability and requirements

**Project name:** DMET Analyzer

**Project home page:**https://sourceforge.net/projects/dmetanalyzer/files/.

**Operating system(s):** DMET Analyzer tool is available for Windows, Linux, and MacOSX operating systems.

**Programming language:** Java

**Other requirements:** Java 1.6.1 Runtime or higher.

**License:** GNU GPL.

**Any restrictions to use by non-academics:** The software is for academic purposes only.

## Endnotes

^a^http://www.affymetrix.com^b^http://www.java.com

## Competing interest

The authors declare that they have no competing interests.

## Authors’ contributions

PHG conceived the main idea of the algorithm and designed the tests. MC leaded the software development process and supervised the design of the algorithm. PHG and MC designed the functional requirements of the software tool. GA implemented the software and performed the software tests. MTD performed medical experiments and participated in the design of the algorithm. MA performed medical experiments. PST and PFT designed the study and performed data interpretation. All authors read and approved the final manuscript.

## Supplementary Material

Additional file 1 Detailed Comparison among DMET Analyzer and relatd softwares. The additional file provides a detailed comparison of DMET-Analyzer with respect to related softwares. Click here for file

Additional file 2 DMET-Analyzer Tutorial. File provides a quick and easy guide to installation and use of DMET-Analyzer. Click here for file
