# Supplementary material for: DMET-Analyzer: automatic analysis of Affymetrix DMET Data
Source: BMC Bioinformatics. 2012 Oct 5;13:258. doi: 10.1186/1471-2105-13-258 (PMC3496574; doi:10.1186/1471-2105-13-258)
Supplement: Additional file 1 — Detailed Comparison among DMET Analyzer and relatd softwares. The additional file provides a detailed comparison of DMET-Analyzer with respect to related softwares. [file 1471-2105-13-258-S1.pdf]

DMET-Analyzer: automatic analysis of Affymetrix DMET Data.
Supplementary Material S1 - Detailed Comparison among DMET Analyzer and relatd softwares

Pietro Hiram Guzzi, *[1] and Giuseppe Agapito[1],and Maria Teresa Di Martino[2],and Mariamena Arbitrio [3], and Pierfrancesco Tassone[2], and Pierosandro Tagliaferri[2], and Mario Cannataro[1],[4]

[1]Department of Medical and Surgical Sciences, Magna Graecia University of Catanzaro, Italy
[2] Magna Graecia University of Catanzaro, Medical Oncology Unit and Tommaso Campanella Cancer Center, Salvatore Venuta Campus
[3] Institute of Neurological Science (ISN-CNR), Roccelletta di Borgia, Italy [4]ICAR-CNR, Rende, Italy

Email: hguzzi*- hguzzi@unicz.it; Giuseppe Agapito - agapito@unicz.it; Maria Teresa Di Martino - teresadm@unicz.it; Mariamena Arbitrio - arbitrio@isn.cnr.it; Pierfrancesco Tassone - tassone@unicz.it; Pierosandro Tagliaferri - tagliaferri@unicz.it; Mario Cannataro - cannataro@unicz.it;

*Corresponding author

1 Related Sofwares

This paper presents a comparison of DMET-Analyzer with three main Genome Association Study tools. Finally a Synoptic table summarizes main differences among tools.

1.1 LDheatmap

LDheatmap (Linkage Disequilibrium heatmap) is a heatmap based graphical display of pairwise linkage disequilibrium between SNPs, freely available as an *R* package. To use *LDheatmap* it is necessary to start *R* from the command line console, typing *R* from the prompt. After loading *R*, it is possible to start *LDheatmap* with the following command: library(”LDheatmap”). The LDheatmap function takes in input a data set that contains information on pairwise *LD* between SNPs in a genomic region, then plots color-coded values of the pairwise *LD* measurements, and returns an object of class ”*LDheatmap*” containing a number of useful information for further analysis. The *LDheatmap* input includes *SNP* genotypes that must be ”genotype” objects created by the ***genotype()*** function from the genetics package, *LD* measurements, or an *LDheatmap* object returned by the function ***LDheatmap()***. In general, *LDheatmap* can visualize any square matrix (with values between 0 and 1) give in input. Other at than display functions, *LDheatmap*

may use the functionality available in *R* in order to perform more complex data analysis or it is possible to save the data to do further analysis with other software. For this reason, it is clear that for using *LDheatmap* is necessary to know the *R* syntax. The following is an example of how to use *LDheatmap*:

1.> library("LDheatmap") (*command for starting LDheatmap from the R prompt*)

2.> data("CEUData") (*loading of the CEU dataset included in the package*)

3.> Heatmap < − LDheatmap(CEUSNP, CEUDist, LDmeasure="r",
title="Pairwise LD in $r \wedge 2$", add.map=TRUE,
SNP.name=c("rs2283092", "rs6979287"),
color=grey.colors(20), name="myLDgrob", add.key=TRUE) (*command for the creation of the Heatmap object and definition of some attributes such as title, colors of the heat map etc*).

1.2 plink

plink is a free open-source line command standalone tool designed to perform genomic data analysis, with greater emphasis on whole genome association data. Furthermore, to help the user into the data analysis, it is possible to download a **GUI** (Graphical User Interface) , in order to use interactively *plink*, through the installation of the *gPlink* package (developed in Java). In addition to the *gPlink* package, it is possible to download and install the *Haploview* package, that can be useful for the annotation, visualization, and storage of the results. plink is a tool that can perform many types of analysis in an efficient manner such as $Hardy\&Weinberg$ eqilibrium tests, missing genotype rates, complete linkage hierarchical clustering, meta analysis, etc. Furthermore it is possible to extend the functionalities through the *R* plugin (for full list of features, see the *plink* manual). To use *plink* in the most efficient way, it is recommended run *plink* form command line. Thus, to start a data analysis in *plink* is necessary to enter the commands in the prompt. The following is a basic example that explains how to use *plink* from the command line to do some basic data analysis. The used dataset, is a toy dataset available in the *plink* web page, in the tutorials section. The command:

plink –file hapmap1 –make-bed –mind 0.05 –out highgeno" (creates a new file that contains individuals with genotyping complete at least 95%. Where the **"–file hapmap1"** option specify the input file where retrieve the data to analyze, **"–make-bed"** option specify to make a binary file, **"–mind 0.05"** option define the threshold below which to discard the results, and **"–out highgeno"** define the name of the results file).

1.3 SimHap GUI

SimHap is a *R* command line package that uses biallelic *SNP* genotype data to impute haplotype frequencies at the individual level. *SimHap* also tests for haplotype associations with outcomes of interest while incorporating the uncertainty around inferred haplotypes into the modeling procedure. *SimHap* allows epidemiological (ie, non-genetic) and both single *SNP* and haplotype association analyses of quantitative Normal, binary, longitudinal and right-censored outcomes under a range of genetic models. *SimHap GUI* provides a graphical user interface to the *SimHap R* package, in order to simplify the uses. *SimHap GUI* guides the user through each step of the analysis process, making it simple to both novice and advanced users. *SimHap GUI* provides an easy way to conduct epidemiological analysis, single *SNP* and haplotype-based association, functions available in *SimHap R* package. Furthermore, *SimHap GUI* provides enhanced functionality such as sophisticated data checking, automated data conversion, and real-time estimations of haplotype simulation progress. *SimHap GUI* is written in Java feature that allows it to work on major operating systems that are compatible with the *Java Virtual* machine. For this reason, *SimHap GUI*requires an installation on the own computer of the *Java Virtual Machine*, *R* Statistics Language and *SimHap R* package to work correctly.

2 A synopsis on Genome Association Study Tools

Table 1: Comparison of DMET-Analyzer and apt-dmet-genotype with respect to some GWAS tools.

Tool	Supported Functions	Native import of DMET data	Part of a package/suite or Standalone program	Cross Platform	Availability of a GUI
DMET-Analyzer	HeatMap Visualization, Statistical Analysis, Search in DB, Correlation with Pharmacogenomic Pathways, Hardy-Weinberg Calculator	YES	Standalone	YES	YES
Plink	Quality Control, Population Stratification Analysis, Association Test	NO	Standalone	YES	YES (in association with gplink)
LDHeatMap	Pairwise linkage disequilibrium analysis	NO	Require the R system	YES, Source codes are provided	Based on R
apt-dmet-genotype	Preprocessing of DMET raw .CEL data	YES	Standalone	YES, Source codes are provided	NO
SimHap Gui	SimHap GUI provides a graphical user interface to the SimHap R package. SimHap epidemiological analysis, single SNP and haplotype-based association	NO	Require the R system	YES	YES, SimHap GUI based on R
