# Supplementary material for: DMET-Analyzer: automatic analysis of Affymetrix DMET Data
Source: BMC Bioinformatics. 2012 Oct 5;13:258. doi: 10.1186/1471-2105-13-258 (PMC3496574; doi:10.1186/1471-2105-13-258)
Supplement: Additional file 2 — DMET-Analyzer Tutorial. File provides a quick and easy guide to installation and use of DMET-Analyzer. [file 1471-2105-13-258-S2.doc]

**DMET-Analyzer** Tutorial

##### Pietro Hiram Guzzi

##### Giuseppe Agapito

##### Mario Cannataro

## Introduction.

DMET-Analyzer, is a novel software tool able to analyze in a quick and easy way data obtained by DMET.

***Availability.***

The latest version of DMET-Analyzer, can be downloaded from <https://sourceforge.net/projects/dmetanalyzer/files/>

In order to install DMET, user should only unzip file and then double click DMETAnalyzer.jar file. Experienced user may also launch this file by the command prompt or shell.

***Requirements.***

DMET-Analyzer is a platform-independent application built in Java. Before starting the analysis with DMET-Analyzer, you will need:

- a system compatible with the last Java Virtual machine SE 6 or higher, and the Java Runtime Environment (JRE) must be installed on your computer.
- a file containing DMET data. Currently supported file extensions include .xls (Excel file organized in columns), txt (tab delimited plaintext).

## Using DMET-Analyzer

### Step 1: Start DMET-Analyzer.

DMET-Analyzer may be launched using the provided launching files: DMET-Analyzer.sh for (Linux or Mac OS X) and DMET-Analyzer.bat (for Windows OS). Alternatively, you can directly launch the program using the Java Virtual Machine with the command java –Xmx2G -jar DMET-Analyzer.jar from the command line, in order to better determine the working memory. In particular the Xmx2G string allocates 2 Gigabytes of memory.


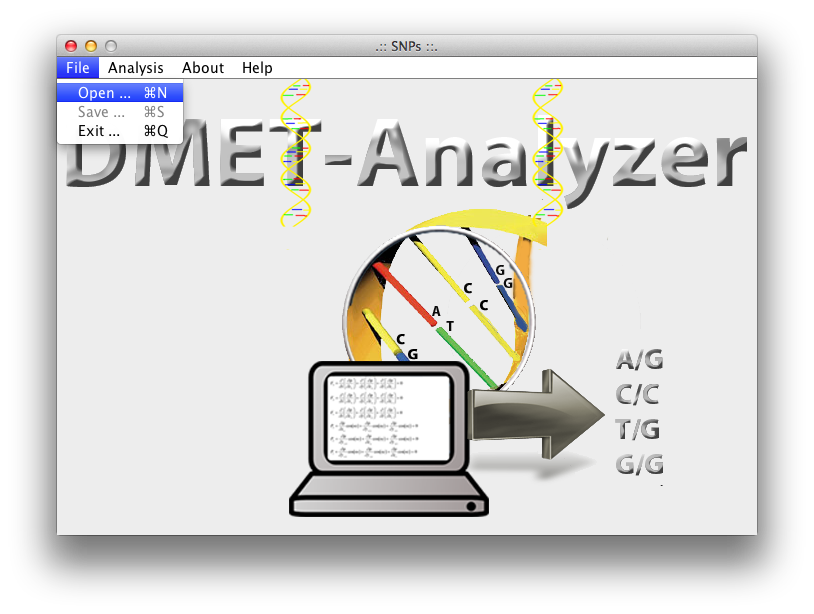


Figure 1. DMET-Analyzer main window.

After launching the program you may open your input data file encoded as *XLS* file or *TXT* file, choosing from the menu the file as shown in Figure 1. Furthermore, if the file contains the class labels (strings specified into the second row), choosing the appropriate file type from the menu, the labels will be visualized such as columns headers in order to simplify the subjects’ selection (*cases vs controls*) see Figure 2.


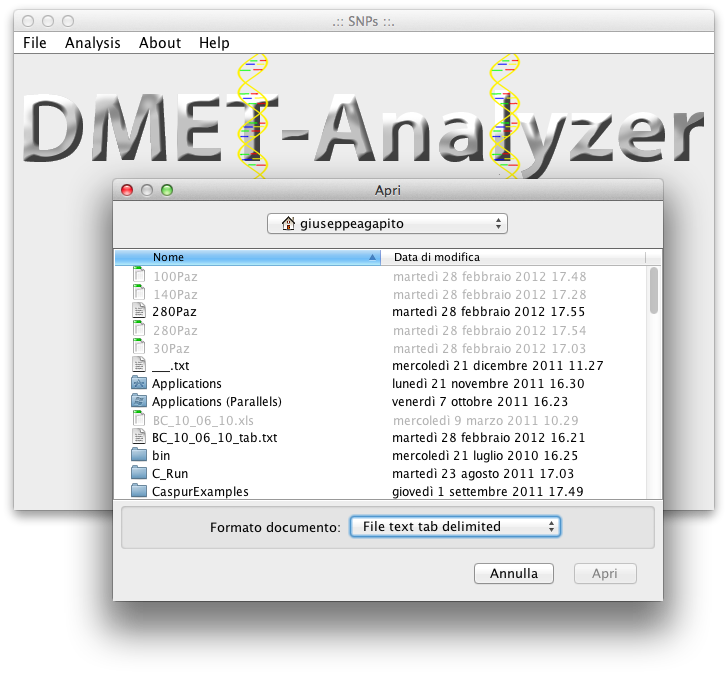


Figure 2. Loading a specific file type, by the menu into the loader module.

Before data visualization, DMET-Analyzer shows user a reminder for the creation of the two classes. The reminder is shown in the Figure 3.


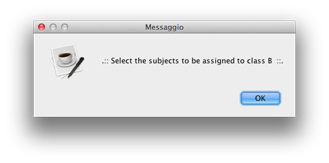


Figure 3. User reminder.


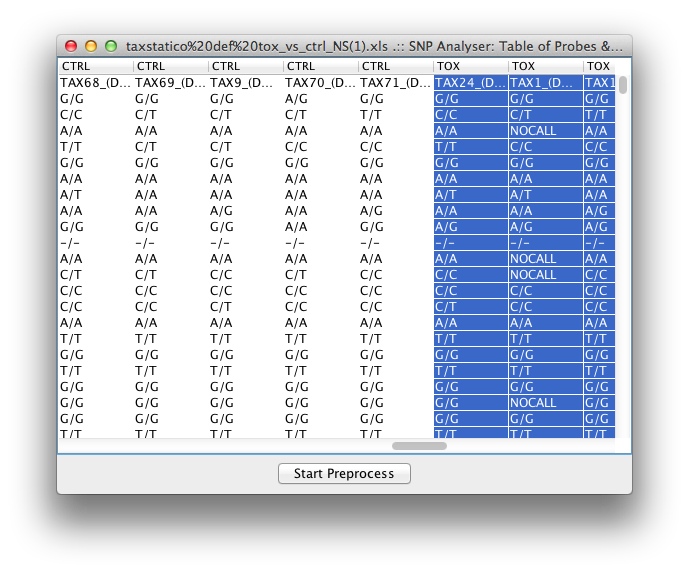


Figure 4. Input visualization and selection.

After loading has been completed, data is shown to the user in a window. Then user may select the classes for each sample by clicking on table columns, shown in Figure 4.

### Step 2: Data preprocessing.

At this point user is asked to assign each sample to the right class through the columns selection. If no columns are selected an error message is displayed, such as shown in Figure 5.


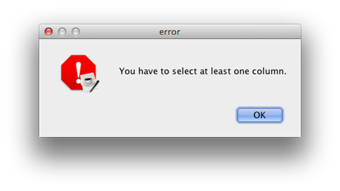


Figure 5. Error message, no columns selected before to start data preprocess.

After, DMET-Analyzer asks to set the names of two classes, if you want to modify the labels that are present in the input file or if you want to assign a class label (in case of missing information in the input file) as shown in Figure 6.


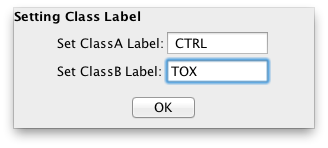


Figure 6. Classes name setting.

Furthermore, in order to simplify the analysis of individual probes, the initial table is reorganized and displayed as a heat map taking into account the subdivision of samples in two classes as depicted Figure 7 in which samples belonging to class B are grouped and moved to the left of the screen.


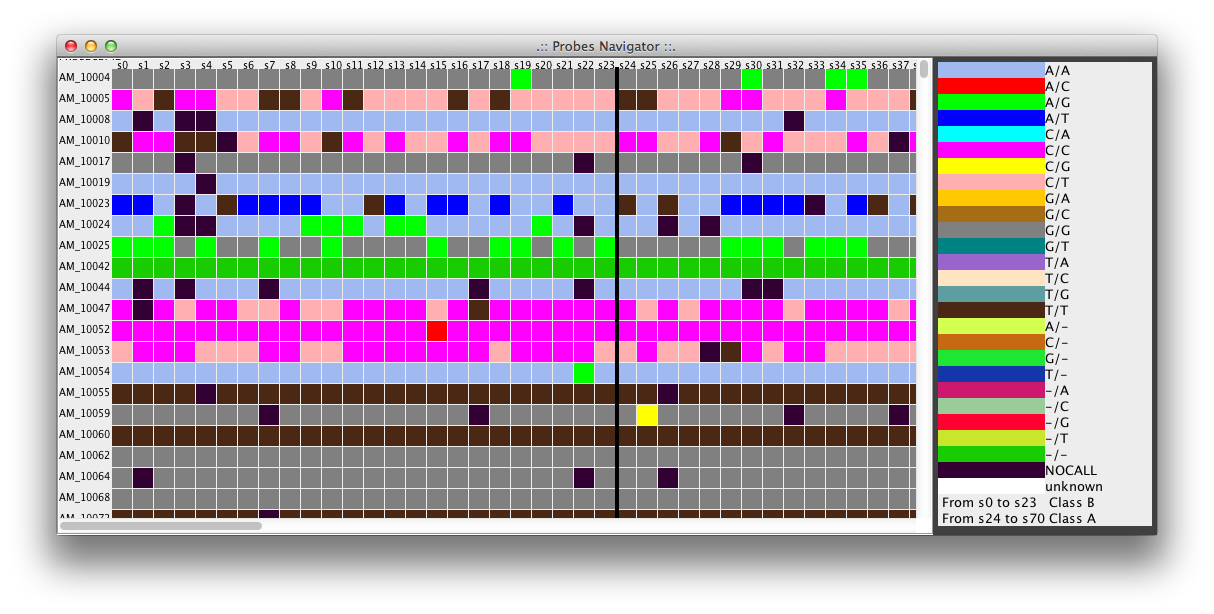


Figure 7. Heat Map probe navigator window.

In parallel, for each probe DMET-Analyzer calculates allele frequencies and shows them in a single table, depicted in Figure 8. Each element (i,j) of this table represents the occurrence of the allele j on probe i. In particular, the figure shows the differences among the frequencies of the alleles with respect to the two classes. Each element (i,j) of this table represents the differences among occurrences in the two classes of the allele j on probe i. This table is interactive because for each probe it is possible to retrieve annotations of all probes by just clicking on the probe’s name (first column). This table is helpful for calculating the Hardy-Weinberg equilibrium. User may browse the Analysis menu or use the keyboard shortcut (“***Commad+I*** ” on a Mac or “***Ctrl+I*** ” on Windows and Linux as shown in Figure 8).


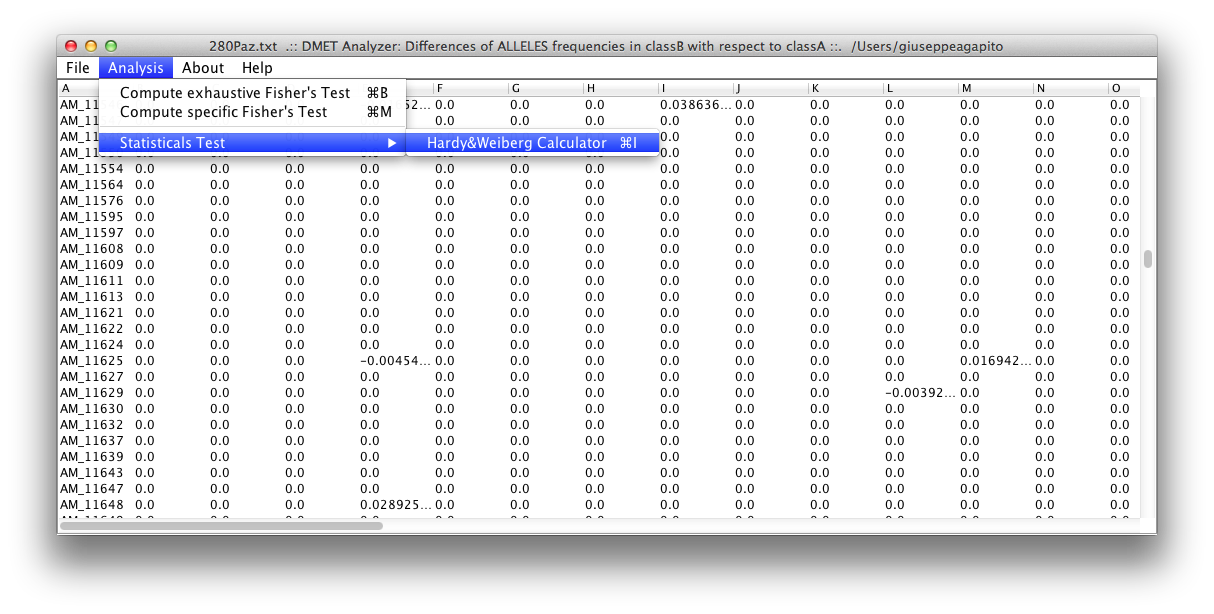


Figure 8. Allele frequencies for each probes.

In this release of DMET-Analyzer, the Hardy and Weinberg equilibrium can be calculate only manually. Users can take advantage of the values contained in the table shown in Figure 8, in order to conduct analysis only on specific probes that users believe to be more interesting. In order to calculate Hardy and Weinberg Equilibrium, users have to type the values in the apposite white text area, see Figure 9, and press ***Calculate***. Finally DMET-Analyzer shows the result of the test.


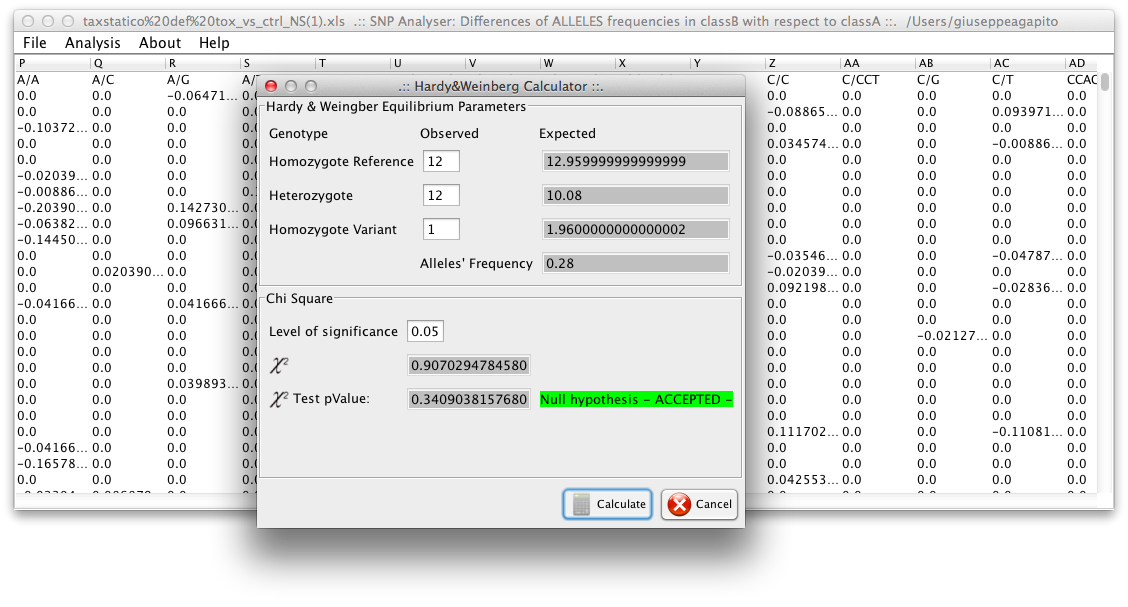


Figure 9. Hardy and Weingber calculator window.

In order to complete the analysis, the user can select the statistical test from the Analysis menu (see Figure 8). By choosing Exhaustive Fisher Test, all the probes will be considered, while by choosing Specific Fisher test DMET-Analyzer will prompt a combo box for selecting a specific probe.

At this point DMET-Analyzer shows a menu from which it is possible to activate statistical corrections for multiple test and to set the threshold of significance by setting the threshold value, as shown in Figure 10.


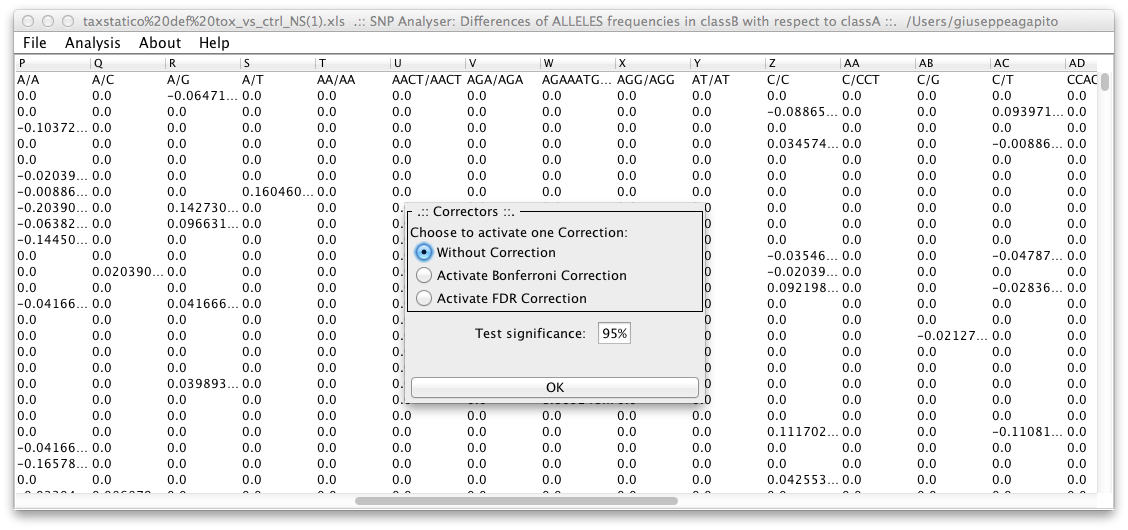


Figure 10. Setting test significance and statistical corrector activation.

### Step 3: Statistical Analysis.

Once the parameters have been set, DMET-Analyzer asks the user if he/she wishes to activate the “Verbose Modality”, to display all the results or only those that are statistically significant, see Figure 11.


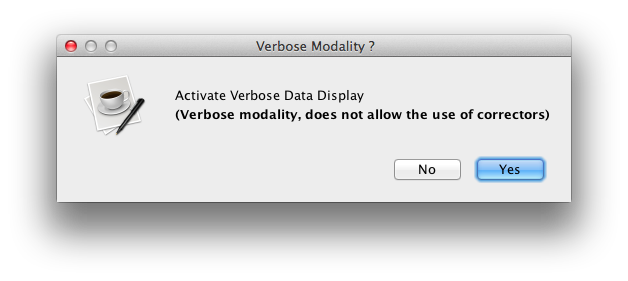


Figure 11. Window that allow the Verbose Modality activation.

In the case of verbose modality, DMET-Analyzer shows all the results, thus it does not allow the use of correctors, showing all the results. Alternatively only the SNPs that are statistically significant are shown. The results are calculated and presented in a separate window using a tabular format as shown in Figure 12. Results may be sorted alphabetically and by p-values. Furthermore clicking on the result panel, it displays a menu that allows saving the results for further analysis (to confront the different results obtained with different subject selection). Note that the number of results are reduced considerably if compared with the number of results obtained with verbose and "*not ALELLE*" modality activated (Figure 14 and Figure 12).


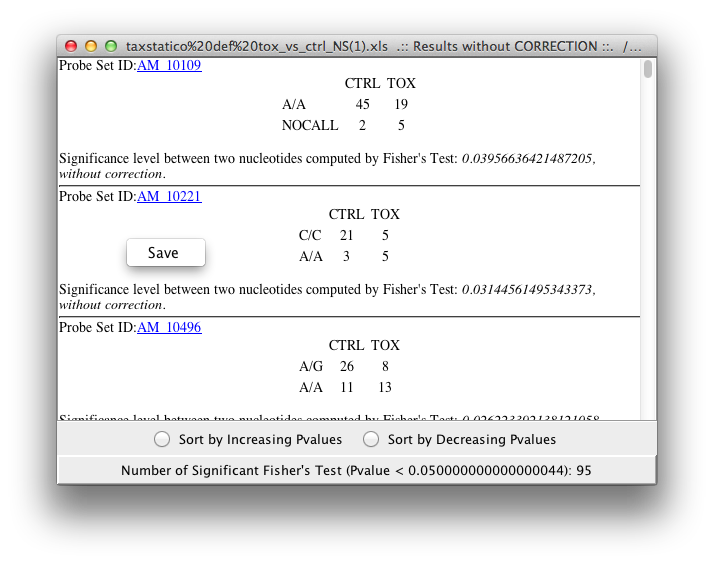


Figure 12. DMET-Analyzer significance of the results and save result menu.

Moreover, it is possible too compute one allele with respect to the other ones (not allele option see Figure 13).


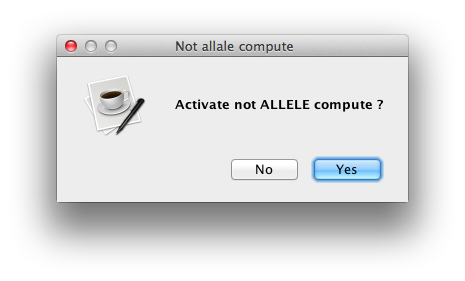


Figure 13. “not ALLELE” compute request of activation.

The results obtained with “not ALLELE” activated are shown in the Figure 14.


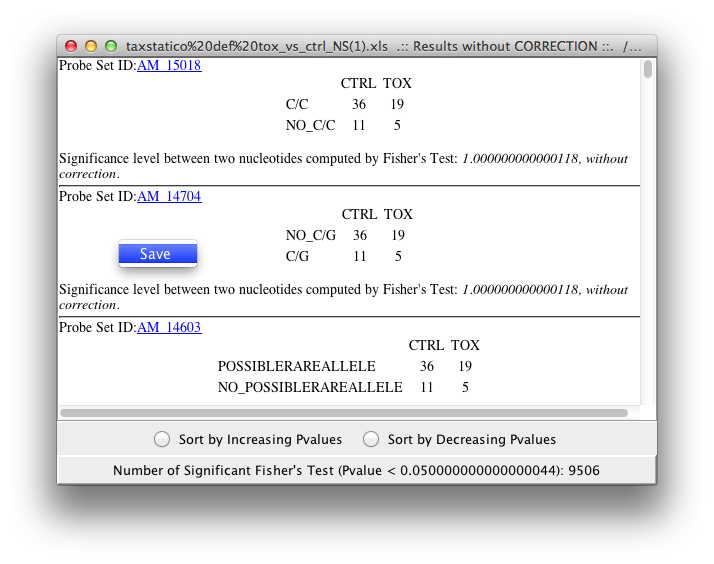


Figure 14. Display verbose result with “not allele” computing.

Each probe present in the result window, is formatted as an hyperlink (Figure 14 and Figure 12), that allows the user to visualize annotations (see Figure 15) and links external databases such as ***dbSNP*** and/or ***PharmGKB*** to retrieve automatically further SNP information, (Figure 16).


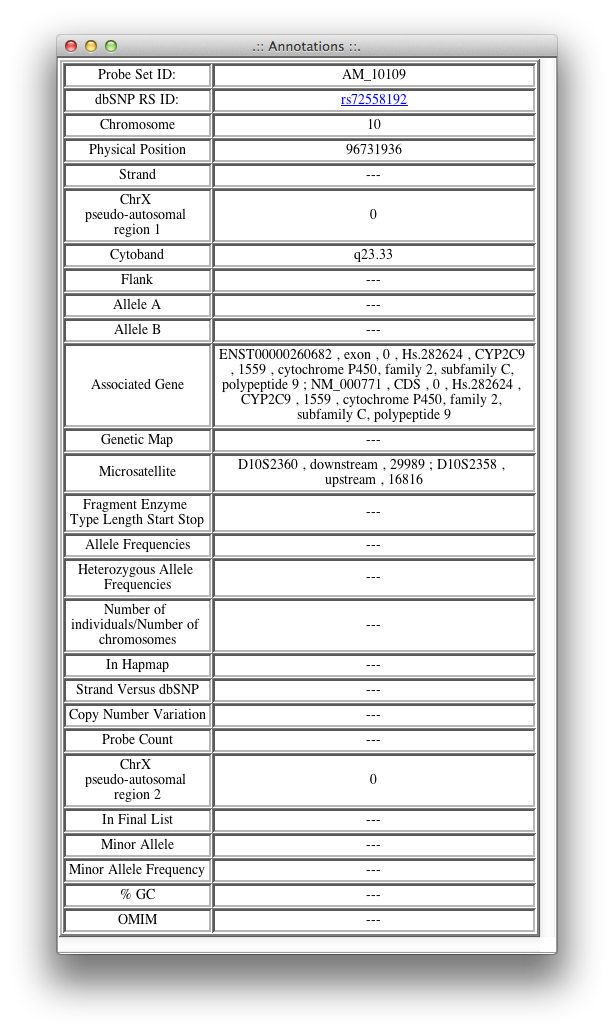


Figure 15. Annotation contained external value (the value in the second column of the second row is used as a key to query external databases) use such identifier to query dbSNP and/or PharmGKB to obtain further information.

| 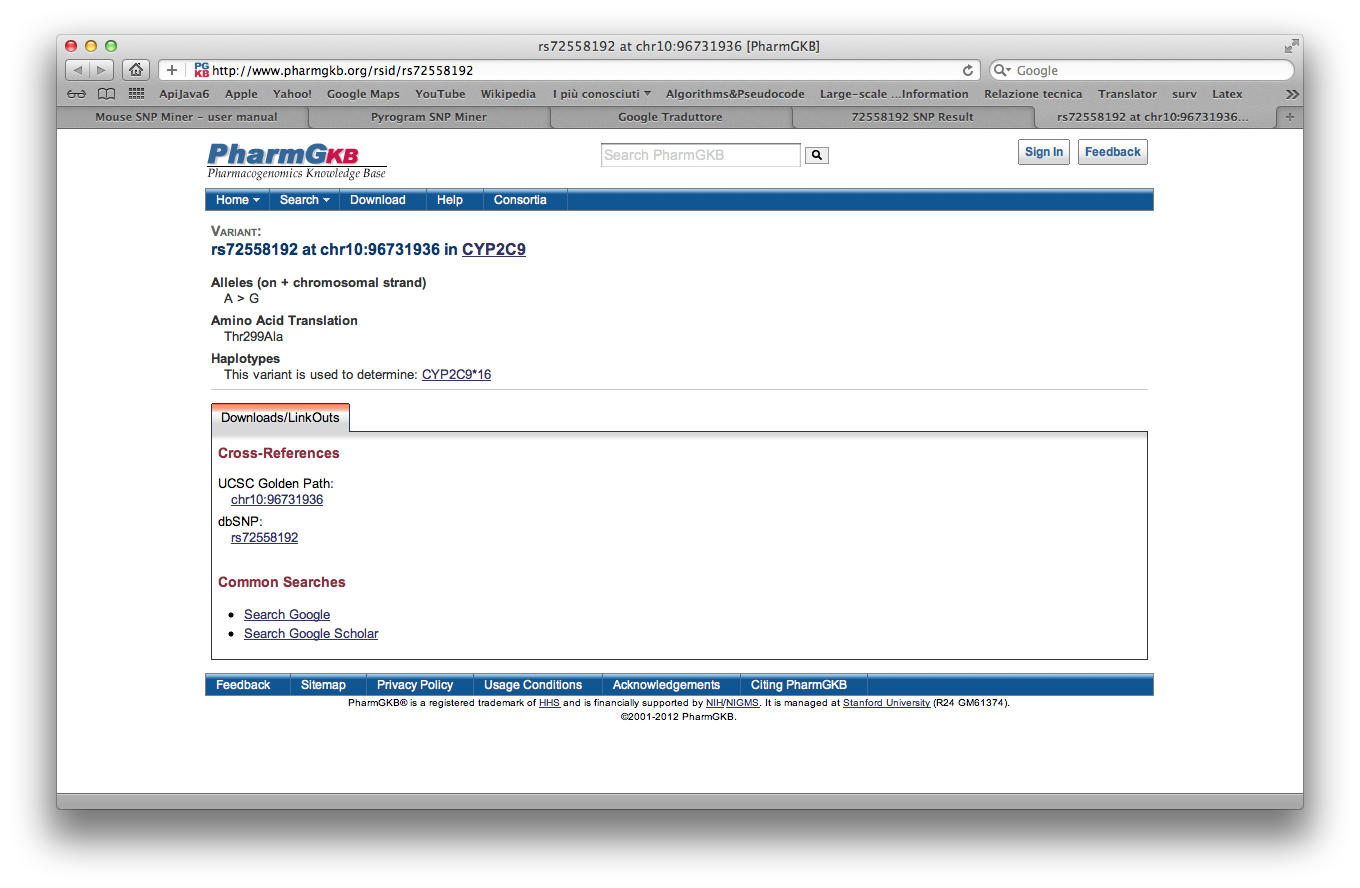  **a. PharmGKB** |
| --- |
| 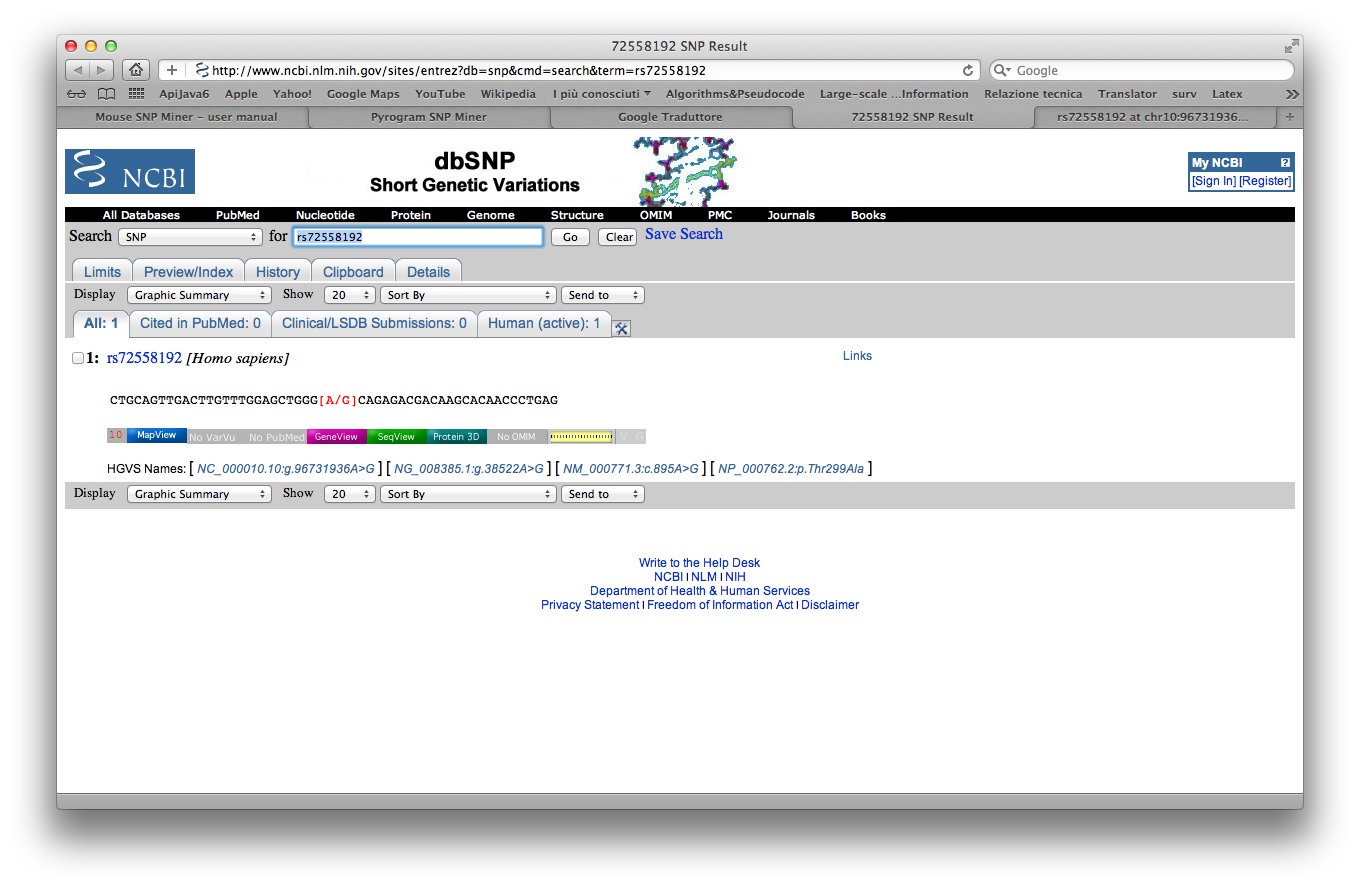  **b. dbSNP** |

Figure 16. External information from dbSNP and PharmGKB, displayed automatically in the user browser.

If the analysis does not produce any results, an error occurs, to find the problem, it is possible to use the Console available in the Help menu (see Figure 2 and Figure 8). In the Console information such as: files loaded, allocated memory size, formatting error in the input file and so on, is displayed, see Figure 17.


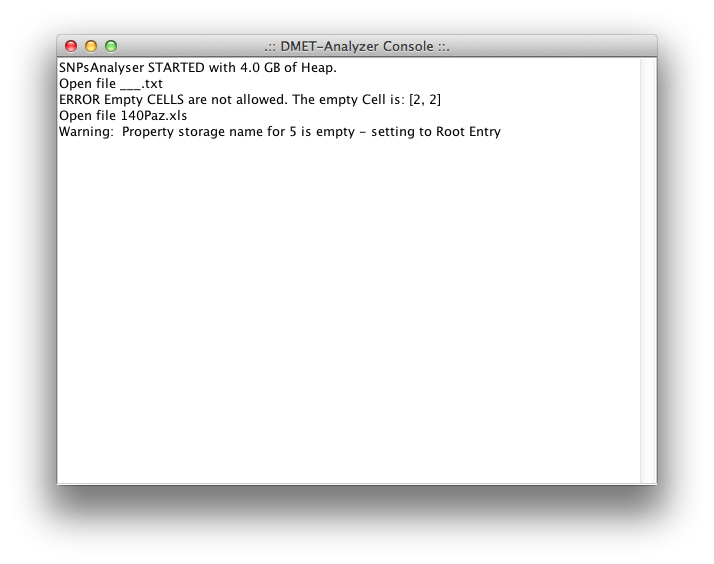


Figure 17. DMET-AnalyzerConsole, with some information on the data analysis.
